# Modulation of the perforant path-evoked potential in dentate gyrus as a function of intrahippocampal *β*-adrenoceptor agonist concentration in urethane-anesthetized rat

**DOI:** 10.1002/brb3.199

**Published:** 2013-12-20

**Authors:** Rebecca L Lethbridge, Susan G Walling, Carolyn W Harley

**Affiliations:** 1 Behavioral Neuroscience, Psychology Department, Memorial University of NewfoundlandSt. John's, NL, A1B 3X9, Canada

**Keywords:** Long-term depression, LTP, NE-LTP, noradrenaline, norepinephrine

## Abstract

**Background:**

*β*-adrenoceptor activation in the hippocampus is sufficient to induce heterosynaptic long-term potentiation of perforant path input to the dentate gyrus. However, in vitro and in vivo studies suggest the plasticity effects of *β*-adrenoceptor activation may vary depending on the level of receptor activation.

**Methods:**

The present experiments use an in vivo model concurrently infusing differing concentrations of the *β*-adrenoceptor agonist, isoproterenol (ISO; 0, 0.1, 1, 10, and 100 *μ*mol/L in aCSF; 1 *μ*L over 12.5 min) in the dentate gyrus, while monitoring changes in the perforant path-evoked potential at the same site.

**Results:**

Long-term depression (LTD) of fEPSP slope was elicited by 0.1 *μ*mol/L ISO. Higher doses did not alter fEPSP slope. Maximal long-term potentiation of the perforant path-evoked population spike (183% >3 h) occurred at 10 *μ*mol/L ISO. Transient depression of spike amplitude occurred at 0.1 *μ*mol/L ISO.

**Conclusions:**

These data demonstrate concentration-dependent effects of *β*-adrenoceptor activation on the perforant path-evoked potential. Long-term depression and long-term potentiation of perforant path-evoked responses are variably elicited as a function of the degree of receptor activation.

## Introduction

The electrophysiological effects of *β*-adrenergic activation on perforant path-evoked potentials in the dentate gyrus have been studied extensively in vitro using the *β*-adrenergic receptor agonist isoproterenol (ISO) (Lacaille and Harley 1985; Dahl and Sarvey 1990; Dahl and Li 1994a), but to date no in vivo recordings with infused ISO have been attempted. This study addresses this issue and characterizes a spectrum of dose–response effects of ISO on both the dentate gyrus—perforant path-evoked field excitatory postsynaptic field potential (fEPSP) slope and population spike.

Previous in vivo studies indicate that *β*-adrenergic receptor-dependent activation in the dentate gyrus reliably recruits potentiation of the perforant path population spike (Harley and Milway 1986; Harley et al. 1989; Washburn and Moises 1989; Kitchigina et al. 1997; Chaulk and Harley 1998; Walling and Harley 2004; Walling et al. 2004; Knight and Harley 2006), while effects on fEPSP slope are more variable with both potentiation or mixed effects including potentiation and depression (Harley and Milway 1986; Chaulk and Harley 1998) or no changes (Washburn and Moises 1989; Walling and Harley 2004; Walling et al. 2004) being reported.

In vitro fEPSP slope potentiation (Lacaille and Harley 1985; Dahl and Sarvey 1989; Pelletier et al. 1994) and population spike potentiation (Lacaille and Harley 1985; Stanton and Sarvey 1985; Dahl and Sarvey 1989; Burgard and Sarvey 1991; Dahl and Li 1994a) have been observed with *β*-adrenoceptor activation, but population spike potentiation is again the more robust of the two effects (Lacaille and Harley 1985; Dahl and Li 1994a,b1994b).

With in vitro activation of *β*-adrenergic activation receptors there is a threshold (∼1 *μ*mol/L ISO) for the occurrence of long-term potentiation (Dahl et al. 1990; Dahl and Li 1994a). In vivo there is also a critical threshold for the long-term population spike potentiation effects using norepinephrine as an activator (estimated synaptic concentration of ∼3 *μ*mol/L) with lower concentrations producing shorter term potentiation (Harley et al. 1996).

Here, four concentrations of the *β*-adrenergic receptor agonist ISO, and a vehicle (aCSF) control were infused adjacent to a recording electrode in the dentate gyrus of the hippocampus in urethane-anesthetized rats. Evoked potentials elicited by single pulse stimulation of the perforant path every 30 sec probed the magnitude of the perforant path fEPSP and the population spike. Evoked potential changes elicited by a 12 min infusion period were followed for 3 h.

## Material and Methods

### Subjects

Male Sprague-Dawley rats (250–400 g; Memorial University of Newfoundland) were used. Rats were housed under a 12:12 h light condition (lights on at 08:00 h) and fed regular rat chow and water ad libitum. All procedures were conducted in accordance with the Canadian Council on Animal Care specifications, and were authorized by the Memorial University Institutional Animal Care Committee.

### Surgery

Rats were anesthetized with urethane (1.5 g/kg, i.p.) and placed in a stereotaxic instrument in the skull flat position and body temperature was monitored and maintained at 37°C by a thermoregulated heating pad (FHC, Bowdoin, ME). Electrode placements were mapped according to the coordinates found in the Paxinos and Watson brain atlas (Paxinos and Watson 1998) for the perforant path (7.2 mm posterior and 4.1 mm lateral from bregma) and for dentate gyrus (3.5 mm posterior and 2.0 mm lateral). A concentric bipolar stimulating electrode (NE-100; Kopf Instruments, Tujanga, CA) was lowered into the perforant path (∼3.0 mm from brain surface). A conjoined electrode/cannula assembly was constructed of a single stainless steel recording electrode (0.5–1 MΩ; FHC Inc.) and a 22-gauge stainless steel guide cannula (Plastics One, Roanoke, VA). The cannula and electrode, secured together with regular epoxy, were aligned so that a 28 gauge injection cannula, when inserted into the guide cannula would sit, ∼25 *μ*m lateral and 50 *μ*m dorsal to the tip of the electrode. This ensured that the concentration delivered at the recording site was as close as possible to the concentration infused. The internal injection cannula (Plastics One) was attached to a solution-filled (ISO in aCSF or aCSF only) autoanalyzer tubing and a dH_2_O-filled 5 *μ*L microsyringe. The total injection (guide and internal cannula) and recording assembly was then slowly lowered into the granule cell layer of the dentate gyrus (∼2.5–3.5 mm from brain surface). The electrode placement was localized to the granule cell layer by monitoring the response to 0.2 ms test pulses delivered to the perforant path and by maximizing the positive-going fEPSP and negative-going population spike.

### Stimulation and recording procedures

Single monophasic square wave test pulses (0.2 ms) were delivered to the perforant path using an interstimulus interval of 30 sec (Neurodata Instruments, New York, NY). The evoked responses were amplified, filtered (0.3 Hz to 3 kHz; P5-11; Grass Instruments, West Warwick, RI), and digitized at a rate of 10 kHz and stored online for analysis.

At the commencement, and at the termination of the recording period, an input–output current intensity series (I/O curve) was determined. This consisted of sampling three evoked responses at interstimulus intervals (ISI) of 10 sec, at each current level from 100 to 1000 *μ*A at 100 *μ*A intervals. On the basis of the initial I/O curve, a current intensity for baseline current stimulation was chosen at the intensity that produced approximately 50% of the maximal population spike.

DataWave software (DataWave Technologies; Loveland, CO) was used to collect waveforms and analysis was performed after the experiments (see Data Analysis and Statistics). Baseline-evoked responses were recorded every 30 sec for at least 1 h before ISO infusion began.

### Procedures for isoproterenol infusion

The drug for infusion, *l*-isoproterenol (Sigma, St. Louis, MO), was made up to four different concentrations (0.1, 1, 10, and 100 *μ*mol/L) in aCSF and kept frozen until use. The solutions were delivered through an electrical syringe pump (Instech, Plymouth Meeting, PA) calibrated to deliver a total volume of 1 *μ*L over 12.5 min. The experiment consisted of a baseline recording (1 h) and an infusion period, after which evoked responses were recorded for an additional 3 h following termination of infusion.

### Histological assessment of placements, and data analysis

Upon termination of recording, anesthetized animals were decapitated and the brains were removed quickly and frozen for cryostat sectioning. Coronal sections (30 *μ*m) were taken and electrode placements were visualized using cresyl violet staining and glycogen phosphorylase histochemistry, as described by Walling et al. (2007). Animals in which electrode or cannula placement occurred outside the dentate gyrus were excluded from analysis.

DataWave software was used to measure the population spike amplitude (first positive peak − first valley) and fEPSP slope (Δ*V*/Δ*t*) for each waveform recorded. The average value for the last 30 min of baseline recording was used to evaluate changes in both population spike amplitude and fEPSP slope for the duration of recording. Measurements were normalized to the mean of the population spike amplitude or fEPSP slope measurement for the 30-min period prior to infusion onset and transformed into 5-min means.

### Within-group analyses

Repeated measures analysis of variance (ANOVA) was used to evaluate the effects of intrahippocampal application of ISO on population spike amplitude and fEPSP slope at each of the concentrations (aCSF, 0.1, 1, 10, 100 *μ*mol/L × time). If significant effects were found, further post hoc analyses were carried out using Fisher's test for Least Significant Differences (LSD) to characterize the differences. Effects were considered significant if the 5-min means after the start of the infusion were significant (*P* < 0.05) from all (6) 5-min means in the 30-min baseline period. A minimum of two consecutive mean calculations were required to be statistically different from baseline. Significance was set at *P* < 0.05.

### Between-group analyses

Two-way repeated measures ANOVAs (groups: aCSF, 0.1, 1, 10, 100 *μ*mol/L × times: 15 min preinfusion and 15, 110, and 180 min postinfusion) were used to assess differences in the effects among varying concentrations of ISO. Again, LSD analysis was employed when the interaction was found to be significant and the criteria level was set at a minimum of *P *< 0.05.

#### Correlations and input/output curves

Pearson product–moment correlations were calculated across all animals in each group for the natural variations in slope–spike relationships at the same time points analyzed in the between-group tests (15 min preinfusion and 15, 110, and 180 min postinfusion). A two-tailed test of significance (*P* < 0.05) was employed. At the conclusion of each experiment (180 min), a second input/output curve was obtained and the results graphed relative to predrug data.

## Results

Histological analysis confirmed correct electrode/cannula placement in 31 rats: 0.1 *μ*mol/L ISO = 6, 1 *μ*mol/L ISO = 6, 10 *μ*mol/L ISO = 6, 100 *μ*mol/L ISO = 6 and aCSF = 7. The mean of the population spike and the EPSP slope during the 30-min baseline period did not differ between groups and so are reported *en totale*. The mean population spike amplitude and the mean EPSP slope measurement for the 30-min baseline period prior to ISO infusion were 3.52 ± 1.53 mV and 4.12 ±1.9 mV/ms, respectively.

### Concentration-dependent effects of intrahippocampal isoproterenol on the perforant path-evoked fEPSP slope

**Figure 1:**
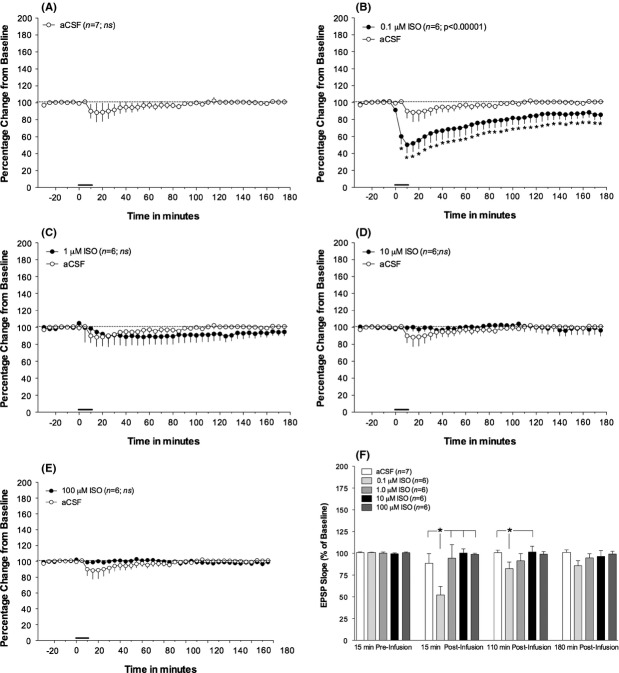
Intrahippocampal infusion of the lowest dose ISO produces a robust *β*-adrenergic receptor-dependent long-term potentiation (LTP) of the perforant path-dentate gyrus fEPSP slope in anesthetized rats. (A) Infusion of vehicle (aCSF;*n* = 7) did not alter the evoked EPSP slope measurement from baseline levels (B) Infusion of the lowest concentration of ISO (0.1 *μ*mol/L) produced a robust, and enduring (>3 h) depression of the fEPSP slope from measurement of the preinfusion (baseline) period. (C–E) Infusion of all other concentrations of ISO (1, 10, and 100 *μ*mol/L) did not significantly alter the fEPSP slope measurement from that of baseline recording. (F) Comparative examination of the effects of intrahippocampal infusion of the varying concentrations of ISO on the perforant path-dentate gyrus EPSP during the recording period. There was a significant concentration × time interaction (*F*_12,78_ = 2.5756; *P* < 0.006). Post hoc analyses revealed that at 15 min postinfusion the lowest ISO concentration (0.1 *μ*mol/L) decreased EPSP slope measurement from vehicle (aCSF)-infused control rats and was also decreased compared to groups infused with all other concentrations of ISO (1, 10, and 100 *μ*mol/L). By 110 min postinfusion the depression of the EPSP slope of the 0.1 *μ*mol/L ISO group was only different from the aCSF group and the 10 *μ*mol/L ISO group. By 180 min postinfusion, no between-group differences in fEPSP slope measurements were observed. Vehicle (aCSF) data are included in all graphs for comparative purposes. Solid bar indicates time and duration of infusion. All data represent means and standard error of the mean. * represents differences in minimum *P* < 0.05 from baseline measurements. See Figure 2 for sample waveforms.

The within-group analysis of the evoked fEPSP slope measurements revealed that only a single concentration of ISO produced long-term effects on the perforant path-evoked fEPSP response. Infusion of 0.1 *μ*mol/L ISO produced a large and persistent depression of fEPSP slope (*F*_41,205_ = 11.746; *P* < 0.00001; *n* = 6; see Fig. 1B). The onset of depression began during the infusion and persisted for the 3-h recording period postinfusion, although diminishing over time. The largest mean decrease, 51% of baseline EPSP slope, occurred 10 min after infusion onset.

Although 1 *μ*mol/L ISO also appeared to produce a small depression of the fEPSP slope, this effect was not significant (*F*_41,205_ = 0.49; *P* < 0.996; *n* = 6; see Fig. 1C). Infusion of aCSF vehicle (Fig. 1A) or other ISO concentrations (10 and 100 *μ*mol/L; Fig. 1D–E) also failed to alter the evoked fEPSP slope.

Between-group analyses of the varying concentrations of ISO revealed a significant interaction (*F*_12,78_ = 2.5756; *P* < 0.006; see Fig. 1F). At 15 min postinfusion, the fEPSP slope of rats receiving 0.1 *μ*mol/L ISO was lower than that observed after any other concentration or aCSF vehicle. By 110 min postinfusion, the fEPSP slope of the 0.1 *μ*mol/L ISO group was still lower than the aCSF and 10 *μ*mol/L ISO groups. Although the pattern of group differences remained similar, by 180 min postinfusion, no differences in fEPSP slope were found among groups.

### Concentration-dependent effects of intrahippocampal isoproterenol on the perforant path-evoked population spike

**Figure 2:**
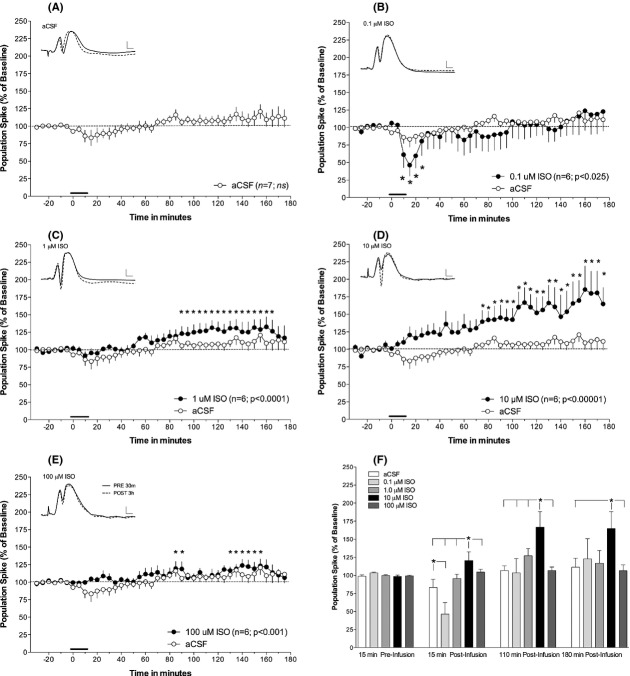
Intrahippocampal infusion of ISO produces a *β*-adrenergic receptor-dependent long-term potentiation (LTP) of the perforant path-dentate gyrus evoked population spike consistent with an inverted dose–response U-curve. (A) Intrahippocampal infusion of vehicle (aCSF) did not alter the population spike amplitude. (B) Infusion of the lowest concentration (0.1 *μ*mol/L) ISO produced a transient population spike depression. (C–E) Infusion of higher concentrations of ISO produced potentiation of the perforant path-dentate gyrus population spike. Potentiation was most robust and enduring after infusion of 10 *μ*mol/L ISO (D), while potentiation after infusions of 1 *μ*mol/L (C) or 100 *μ*mol/L (E) ISO were delayed or fluctuated throughout the recording period. (F) Between-group analysis demonstrated a significant concentration × time interaction (*F*_12,78_ = 2.4123; *P* < 0.01). Post hoc assessment revealed that 15 min postinfusion there was a significant depression of the population spike amplitude in rats in the 0.1 *μ*mol/L group compared to rats in the aCSF infused control group. Furthermore, rats receiving 10 *μ*mol/L ISO infusions had increases in spike amplitude exceeding that of all other groups. This effect on spike amplitude persisted at 110 min, but by 180 min postinfusion the spike amplitude increases in the 10 *μ*mol/L group was only elevated over aCSF-and 100 *μ*mol/L ISO infused rats. *Insets*: representative waveforms of rats infused with ISO 30 min preinfusion (solid line), and 180 min post (dashed line) infusion. Vehicle (aCSF) data are included in all graphs for comparative purposes. Solid bar indicates time and duration of infusion. Scale bar represents 2 mV and 2 ms. All data represent means and standard error of the mean. * represents differences in minimum *P* < 0.05 from baseline measurements.

Graphed data for the intrahippocampal infusions of four concentrations (0.1, 1, 10, and 100 *μ*mol/L) of ISO and the aCSF vehicle on the dentate gyrus evoked population spike are presented in Figure 2. Infusion of the aCSF vehicle (*n* = 7) did not alter the amplitude of the evoked population spike (Fig. 2A).

Repeated measures analysis of the effects within each group revealed a concentration-dependent effect on the evoked population spike. First, infusion of the lowest concentration of ISO, 0.1 *μ*mol/L (*n *= 6) produced a transient depression of the dentate gyrus evoked population spike from baseline responses (*F*_41,205_ = 1.555, *P* < 0.025; see Fig. 2B). Post hoc analysis revealed that this decrease began at the termination of infusion and was maximal at 46% of the baseline spike amplitude. The depression lasted for four 5-min samples (20 min) and then returned to baseline.

The remaining concentrations of intrahippocampal ISO (1, 10, and 100 *μ*mol/L) produced a potentiation of the perforant path—dentate gyrus evoked population spike (see Fig. 2C–E). The potentiation observed at the 1 *μ*mol/L concentration (*F*_41,205_ = 3.3424; *P* < 0.0001; *n* = 6) began following infusion termination and was significantly different ∼75 min later with a maximal mean potentiation from baseline of 133%. The response returned to baseline before 3 h of recording had elapsed. Application of ISO at 10 *μ*mol/L concentration produced the largest and most enduring increases in population spike amplitude (*F*_41,205_ = 4.9210; *P* < 0.00001; *n* = 6). The potentiation began during infusion and was significantly different ∼60 min following infusion termination. The mean maximal increase was 185% and persisted for the duration of the 3-h recording period. Finally, intrahippocampal infusion of the 100 *μ*mol/L concentration produced a small and variable potentiation of the evoked population spike (*F*_41,205_ = 1.97; *P* < 0.001; *n* = 6), with the first significant increase at ∼70 min postinfusion but then returning to baseline, a second elevation occurred 2 h later and lasted 35 min, returning to baseline prior to termination of recording. The maximal mean increase occurred in the second period of potentiation and was 124% of baseline.

A two-way repeated measures ANOVA (concentration × time at 15 min preinfusion and 15, 110, and 180 min postinfusion) was used to assess between-group effects of varying ISO concentrations. A significant interaction was found for the effects of varying concentrations of ISO on the population spike amplitude (*F*_12,78_ = 2.4123; *P* < 0.01). Post hoc analysis showed no differences between groups in the 15-min period prior to infusion; however, 15 min after the start of the infusion of 0.1 *μ*mol/L ISO, the population spike was depressed compared to aCSF-infused control rats (see Fig. 2F). The population spike amplitude in rats receiving 10 *μ*mol/L ISO was greater than that of rats receiving infusions of either aCSF or any other ISO concentration. By 110 min after the start of infusions, the spike amplitude of rats infused with 10 *μ*mol/L was still elevated, compared to all other groups. At 180 min after infusion, the conclusion of recording, the population spike amplitude of the 10 *μ*mol/L group was greater than that of both the aCSF-infused control rats and rats receiving an infusion of the highest concentration of ISO (100 *μ*mol/L).

### Effects of intrahippocampal isoproterenol on fEPSP slope–spike coupling in the dentate gyrus

Input–output curves for the 10 levels of stimulation were taken prior to baseline and at the termination of recording (data not shown). The only significant change (*P* < 0.05) occurred in the 10 *μ*mol/L ISO group in which there was a leftward shift in the input–output relationship. This shift reflected the occurrence of larger spikes postinfusion for the same stimulation levels more than 3 h after infusion initiation, a result consistent with the long-term increase in spike size with 10 *μ*mol/L ISO.

### fEPSP slope/population spike correlations

**Figure 3:**
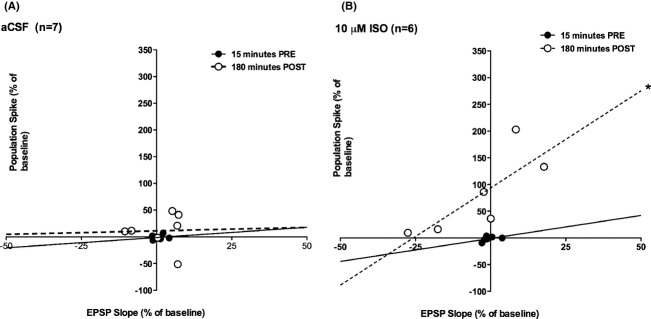
The correlational fEPSP slope and population spike relationship before and after infusion of vehicle (aCSF) or 10 *μ*mol/L ISO. No relationship was found in any group during the preinfusion period (solid line in A and B for examples). (A) No changes were found in the fEPSP slope and population spike correlation after infusion of aCSF at 180 min after infusion (dashed line). (B) Infusion of 10 *μ*mol/L ISO produced a positive correlation between fEPSP and population spike at 110 min (not shown; *P* < 0.005) and 180 min (*P* < 0.05; dashed line) after the infusion.

In the majority of granule cells norepinephrine produces a direct increase in membrane resistance that is mediated by *β*-adrenoceptors (Lacaille and Schwartzkroin 1988). We hypothesized that effective *β*-adrenoceptor activation should increase the EPSP initiation of cell firing. Slope-spike correlations were examined to address this question. There were no significant correlations between the fEPSP slope and spike size prior to infusion. Immediately after infusion (postinfusion onset 15 min) there was a significant positive correlation (*r* = 0.76, *P* < 0.05) in the aCSF group that was not sustained (*r* = 0.52, *ns* at 180 min; see Fig. 3A). In contrast, the 10 *μ*mol/L ISO group exhibited significant positive correlations between EPSP field slope and population spike size at 110 min (*r* = 0.94, *P* < 0.005; not shown) and 180 min (*r* = 0.80, *P* < 0.05) postinfusion, the last two time points measured (see Fig. 3B). No other group showed significant correlations between fEPSP slope and population spike size postinfusion.

## Discussion

### Effects of intrahippocampal ISO infusion on the perforant path-dentate gyrus fEPSP slope

The present pattern of results reveals a *β*-adrenoceptor-induced long-term depression (LTD) of the perforant path-evoked fEPSP in the dentate gyrus at the lowest level of receptor activation in this study (0.1 *μ*mol/L). A weaker level of depression was also seen with the next higher dose (1 *μ*mol/L). Winson and Dahl (1985) using ISO iontophoresis in the mid-dendritic layer reported depression of the perforant path fEPSP; however, fEPSP depression occurred with all noradrenergic agents applied.

In vitro exclusively lateral perforant path activation paired with 1 *μ*mol/L ISO produces a LTD of the fEPSP, while medial perforant path pairing initiates long-term potentiation at the same concentration (Dahl and Sarvey 1990). However, in this study, no attempt was made to isolate lateral perforant path fibers. Stimulation coordinates were selected for optimal generation of a medial perforant path population spike. The stimulating electrode placement was consistent across all concentrations. Isoproterenol at the three higher concentrations had no significant effect on the fEPSP.

The low-concentration *β*-adrenoceptor-elicited fEPSP LTD seen here did not require sustained intermediate frequency LTD stimulation as is typically needed to induce LTD. In vitro *β*-adrenoceptor LTD in the lateral perforant path has been shown to be both *β*-adrenoceptor and NMDA-receptor dependent (Dahl and Sarvey 1989 1990). It will be of interest to examine NMDA dependence of low concentration ISO-induced LTD.

### Effects of intrahippocampal ISO infusion on the perforant path-dentate gyrus population spike

The lowest concentration of ISO produced a transient depression of the population spike. This did not temporally follow fEPSP LTD at the same ISO concentration and the two were not correlated. Winson and Dahl (1985) found a depression of population spike amplitude with ISO iontophoresis, which did not outlast the period of iontophoretic current. Their population spike depression effect was *β*-adrenoceptor receptor selective. With locus coeruleus electrical stimulation they observed spike potentiation and EPSP depression, although EPSPs had to be monitored in the dendritic layer to detect the EPSP change.

The largest, and most enduring (>3 h) potentiation of population spike amplitude seen here occurred at an intermediate concentration of 10 *μ*mol/L ISO. The highest concentration used did not produce long-term potentiation and even the transient potentiation seen was small.

All higher concentrations of ISO produced some degree of *β*-adrenoceptor-mediated population spike potentiation as has been reported in the majority of studies of noradrenergic modulation of the perforant path input to dentate gyrus whether in vivo or in vitro. The long-lasting potentiation effects here increased over time following infusion. Late developing kinetics for norepinephrine-induced long-term potentiation (NE-LTP) in dentate gyrus has been reported previously with LC activation (Walling and Harley 2004; Reid and Harley 2010). The mechanism behind this gradually increasing potentiation profile is not known. That such long-term potentiation is not simply a generalized disinhibition effect is demonstrated by the requirement that locus coeruleus activation must be paired with perforant path activation for its occurrence (Reid and Harley 2010). Transient disinhibition, however, is reliably associated with locus coeruleus activation (Brown et al. 2005).

### Increased EPSP-spike coupling

In the dentate gyrus, frequency-induced long-term potentiation (LTP) frequently results in a leftward shift of the EPSP slope-population spike (E-S) relationship (Bliss and Lomo 1973; Kairiss et al. 1987). This leftward shift has also been found in NE-LTP, induced by endogenous NE release in awake rats (Walling and Harley 2004). In this study, the optimal LTP-inducing concentration of ISO (10 *μ*mol/L) also produced a leftward shift in the E-S relationship and this concentration was further associated with a significant correlation between EPSP slope variation and spike variation. While the correlation effect may be partially related to the larger range of spikes seen at this concentration, both the significant correlation and the leftward shift can be parsimoniously accounted for by a *β*-adrenoceptor-mediated increase in granule cell membrane resistance (Lacaille and Schwartzkroin 1988).

### β-adrenoceptor effects as related to locus coeruleus activation

The present results suggest that the *β*-adrenoceptor effects on long-term plasticity of either the fEPSP or the population spike are not linearly related to dose. Intermediate, rather than maximal, doses appear to provide the clearest effects. This is consistent with a reported inverted U-curve for norepinephrine-associated arousal on neuronal activity and/or behavior and may provide an underpinning of such effects in the dentate gyrus. In previous in vivo work all of the plasticity effects of locus coeruleus activation on the dentate gyrus-perforant path-evoked potential, whether in urethane-anesthetized (Harley and Milway 1986; Harley et al. 1989) or awake rats (Kitchigina et al. 1997; Walling and Harley 2004) could be blocked by the *β*-adrenoceptor antagonist propranolol.

Future studies will be required to delineate the substrates of the concentration effects observed. Varying concentrations of ISO may recruit differing proportions of *β*-adrenoceptor subtypes and the predominant localization of the *β*-adrenoceptor subtypes recruited may also differ. Higher concentrations may also be less selective for *β*-adrenoceptors. The main point of interest of the present experiments is that enduring, and differing, plasticity effects are recruited by different levels and/or patterns of *β*-adrenoceptor activation. The local modulation of norepinephrine levels is likely to play a critical role in recruitment of plasticity, at least within the dentate gyrus.

### Naturalistic modulation of dentate gyrus plasticity

The present patterns of results are consistent with independent *β*-adrenoceptor modulation of both synaptic input and cell excitability. Previous naturalistic investigations of novelty exploration, which activates the locus coeruleus (Vankov et al. 1995), together with monitoring of the perforant path-evoked potential, have revealed both an increase in cell excitability (Kitchigina et al. 1997) and a decrease in synaptic input (Moser 1996). A *β*-adrenoceptor antagonist prevented the increase in cell excitability (Kitchigina et al. 1997). The depression of synaptic input was not assessed pharmacologically (Moser 1996). Both forms of plasticity can be input selective (see for example Frick and Johnston 2005). Selective dendritic excitability changes have been proposed as another potential underpinning of learning and memory circuit changes (Frick and Johnston 2005; Reid and Harley 2010).

This study suggests that differences in norepinephrine release levels will recruit varying changes in granule cell information processing through *β*-adrenoceptor modulation, which has an optimal window for enduring plasticity effects. These in vivo infusion experiments also reveal a novel *β*-adrenoceptor-mediated LTD effect on glutamatergic synaptic signaling in dentate gyrus.
